# Histoplasmosis, heart failure, hemolysis and haemophagocytic lymphohistiocytosis

**DOI:** 10.11604/pamj.2019.32.43.14954

**Published:** 2019-01-23

**Authors:** Nitin Gupta, Kutty Sharada Vinod, Ankit Mittal, Aswin Pius Ajay Kumar, Arvind Kumar, Naveet Wig

**Affiliations:** 1 Department of Medicine, All India Institute of Medical Sciences, New Delhi, India

**Keywords:** Pulmonary arterial hypertension, immunocompetent, pancytopenia

## Abstract

Histoplasmosis is an endemic mycosis with global distribution, primarily reported in immunocompromised individuals. A 29-year old immunocompetent male presented with fever, hepatosplenomegaly and pancytopenia. His peripheral blood showed features suggestive of intravascular hemolysis and echocardiography showed features suggestive of pulmonary arterial hypertension. Bone marrow showed yeast with morphology suggestive of *Histoplasma capsulatum*. Further investigations revealed hyperferritinemia, hypofibrinogenemia and increased triglycerides. With a diagnosis of progressive disseminated histoplasmosis with secondary Haemophagocytic lymphohistiocytosis, he was successfully treated with amphotericin B followed by itraconazole. We report this case to highlight the atypical and rare manifestations of histoplasmosis.

## Introduction

Histoplasmosis is an endemic mycosis caused by the dimorphic fungi, *Histoplasma capsulatum*. The mode of infection is by inhalation of microconidia that are usually found in bird or bat guano containing soil. In immunocompetent individuals, such acute infection resolves on its own while the disease may be limited to lungs or disseminate to liver, spleen and bone marrow in immunocompromised [1]. Definite diagnosis of histoplasmosis is made by demonstrating fungi on microscopy or cultures. Cultures may take up to six weeks to become positive and can lead to a significant delay in diagnosis. The treatment of choice is amphotericin B followed by oral itraconazole or itraconazole alone depending upon severity and extent of disease [2]. We report a case of histoplasmosis in a young immunocompetent male with atypical presentations.

## Patient and observation

**Figure 1:**
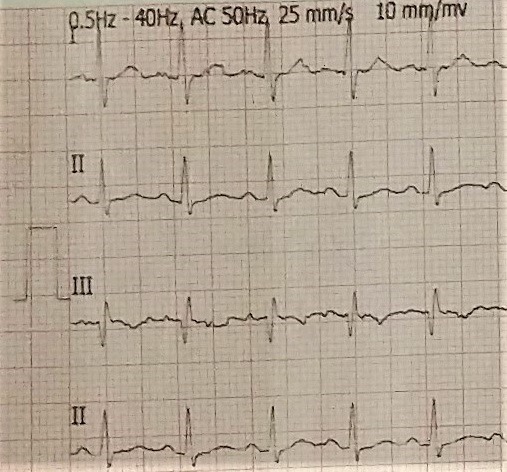
ECG showing S1Q3T3 pattern

A 29-year old male patient from Delhi with no known prior comorbidities presented with fever and dry cough for 40 days and progressively worsening dyspnea for 15 days. This was associated with loss of appetite and loss of weight (5kg in 40 days). On general physical examination, patient was febrile, pulse rate was 134/min, SpO_2_ on room air was 92%, respiratory rate was 36/min and blood pressure was 110/70 mm of Hg. Pallor was present but there was no icterus, cyanosis, pedal edema or lymphadenopathy. On systemic examination, moderate hepatosplenomegaly was present. Chest was bilaterally clear. On initial laboratory investigation, he was found to have pancytopenia (Haemoglobin-8.4/dl, Platelet count-79,000/μl, total leucocyte count-3900/μl). Malarial parasites were not seen on peripheral smear and quantitative buffy coat examination. Blood and urine cultures were sterile. Serology for HIV 1 &2 was non-reactive. Antibodies against rk-39 were negative. Widal titre was < 1:40 for typhoidal O, typhoidal H and paratyphoidal H antigen. Peripheral smear showed presence of schistocytes with a corrected reticulocyte count of 1.8%. Lactate dehydrogenase (LDH) was found to be elevated (579 IU/l) and direct coomb’s test was positive. Vitamin B12 and folate levels were normal. Electrocardiogram (ECG) showed right bundle branch block with S1Q3T3 pattern (Figure 1). B type natriuretic peptide levels (211.6 pg/ml) were elevated. Echocardiography showed moderate pulmonary arterial hypertension (Pulmonary artery systolic pressure- 43+10 mm of Hg), moderate tricuspid regurgitation and prominent right atrium and ventricles. Left ventricle ejection fraction was 70%. Minimal pericardial effusion was also noted. CT pulmonary angiogram was normal. USG Venous Doppler of all four limbs were normal.

**Figure 2:**
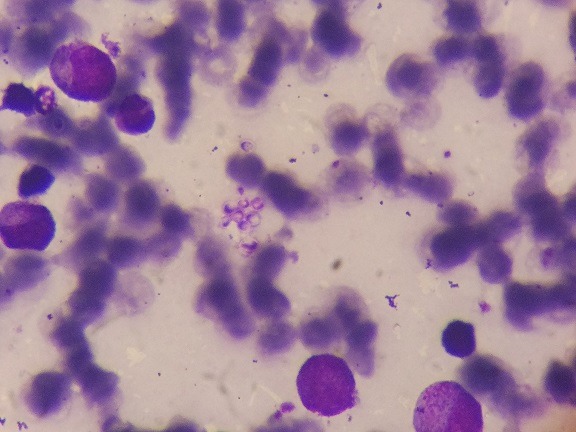
Bone marrow aspirate showing small extracellular cellular yeast cells with a false appearance of capsule around them

Bone marrow aspirate showed mild relative erythroid hyperplasia with both intracellular and extracellular yeast with morphology suggestive of *Histoplasma capsulatum* (Figure 2). Histoplasma serology however was negative. CD4 count was 454/mcl (39.9%). IgA, IgG and IgM levels were normal. Anti-nuclear antibody (ANA) and Anti ds DNA were negative. Fibrinogen levels (< 150mg/dl) were decreased. Triglycerides (304 mg/dl) and ferritin levels (> 2,000ng/ml) were increased. Patient was started on intravenous liposomal amphotericin B. His fever resolved on day 4 of liposomal amphotericin B. His dyspnea, however resolved on day 10 of liposomal amphotericin B. On day 13, he was shifted to itraconazole therapy. Follow up platelet count and total leucocyte count after 12 days of therapy was 1.8 lakh/cu.mm and 4500/cu.mm respectively. The hemoglobin at three weeks after the initiation of therapy was 9.4g/dl. No blood was transfused during or after the hospital stay. Follow up echocardiography on day 5 of amphotericin B revealed mild pulmonary arterial hypertension (pulmonary artery systolic pressure- 36+ 10mm of Hg) and trivial tricuspid regurgitation. Right atrium and right ventricles were normal in size. He resumed his normal daily activities within a week of discharge. He continued to take itraconazole for four months and discontinued on his own. He returned to follow up one year later from the time of discharge for routine checkup. His complete blood count was normal (hemoglobin- 14.6g/dl, total leucocyte count- 7430/ cu.mm, platelet count- 3 lacs/ cu.mm). 2D echocardiography showed a pulmonary artery systolic pressure of 31+ 10 mm of Hg and trivial tricuspid regurgitation.

## Discussion

Histoplasmosis has widespread distribution in the western world but it is also endemic in many parts of South-East Asia. It has been reported in India since the 1950s with variable histoplasmin sensitivity rates (0-12.3%) [3, 4]. Its spread and increasing incidence has largely been attributed to the HIV pandemic and increasing use of immunosuppressive agents in transplant recipients and patients with rheumatological disorders. However, cases have been reported time and again in immunocompetent individuals. In a review by Kathuria *et al.* 61 cases of histoplasmosis in immunocompetent adults was reported between 1995 and 2011 from India [4]. Three large studies from Delhi and South India reported 80 cases of disseminated histoplasmosis in ten years. Most of the cases were reported from the Gangetic delta [5]. Histoplasmosis can also get complicated with secondary Haemophagocytic lymphohistiocytosis (HLH) as a result of a cytokine storm due to an overwhelming activation of lymphocytes and macrophages. This condition may be rapidly fatal if the cause is not discovered and treated adequately. In a review by Sonavane *et al.*, thirty-eight cases of Histoplasmosis associated HLH have been reported worldwide, of which eight cases were from India. Most cases have been reported in the immunocompromised hosts [6]. Anemia in histoplasmosis is common and is mostly because of the bone marrow involvement and/or rarely due to secondary HLH. However, the case described above also had a component of coomb’s positive hemolytic anemia. To the best of our knowledge, only two such cases have been reported but the pathophysiology of such an occurrence is still not clear [7, 8].

Another important clinical feature that our patient had was pulmonary arterial hypertension. The patient had acute worsening in shortness of breath and the ECG showed S1Q3T3 pattern. CT pulmonary angiogram showed no abnormality. We could not explain pulmonary hypertension in our patient as he was asymptomatic prior to this presentation and we could not find any reports that linked histoplasmosis with pulmonary hypertension. However, an interesting finding was noted in our patient. With successful treatment, there was a significant reduction in pulmonary artery systolic pressure (53mm of Hg to 41mm of Hg) on follow up echocardiography. The diagnosis in our patient was confirmed by spotting the typical morphology of histoplasmosis on giemsa staining of bone marrow aspirate. The sensitivity of direct demonstration and culture in progressive disseminated histoplasmosis is 76% and 74% respectively [1]. For disseminated histoplasmosis, amphotericin B is given for 7-14 days followed by oral itraconazole for up to a year. Our patient stopped his treatment after four months of initiation. Considering, he was asymptomatic even after eight months of discontinuation and wasn’t willing to start treatment again, we decided to follow him up closely. Management of secondary HLH requires treatment of the primary disease; however, the chemoimmunotherapy for HLH including steroids, etoposide and cyclosporine may be required in certain cases [9].

## Conclusion

Histoplasmosis is increasingly being reported from the Indian subcontinent, especially in individuals without any risk factors for the disease. We report this case to highlight the atypical and rare manifestations that can be associated with this disease.

## Competing interests

The authors declare no competing interests.

## References

[1] Hage CA, Azar MM, Bahr N (2015). Histoplasmosis: up-to-date evidence-based approach to diagnosis and management. Semin Respir Crit Care Med.

[2] Wheat LJ, Freifeld AG, Kleiman MB (2007). Clinical practice guidelines for the management of patients with histoplasmosis: 2007 update by the Infectious Diseases Society of America. Clin Infect Dis.

[3] Wheat LJ, Azar MM, Bahr NC (2016). Histoplasmosis. Infect Dis Clin North Am.

[4] Kathuria S, Capoor MR, Yadav S (2013). Disseminated histoplasmosis in an apparently immunocompetent individual from north India: a case report and review. Med Mycol.

[5] De D, Nath UK (2015). Disseminated Histoplasmosis in immunocompetent individuals- not a so rare entity, in India. Mediterr J Hematol Infect Dis.

[6] Sonavane AD, Sonawane PB, Chandak SV (2016). Disseminated Histoplasmosis with haemophagocytic lymphohistiocytosis in an immunocompetent Host. J Clin Diagn Res.

[7] Chang Y-T, Huang S-C, Hu S-Y (2010). Disseminated histoplasmosis presenting as haemolytic anaemia. Postgrad Med J.

[8] Chejara RS, Nawal CL, Agrawal MK (2016). Progressive Disseminated Histoplasmosis with Coomb’s positive hemolytic Anemia in an immunocompetent Host. J Assoc Physicians India.

[9] Townsend JL, Shanbhag S, Hancock J (2015). Histoplasmosis-induced hemophagocytic syndrome: a case series and review of the literature. Open Forum Infect Dis.

